# Using a multidisciplinary approach to reveal decision-making capacity within acute care for an individual with aphasia

**DOI:** 10.1177/2050313X211027098

**Published:** 2021-07-09

**Authors:** Ruth Maxwell, Michelle O’Brien, Deirdre O’Donnell, Lauren Christophers, Thilo Kroll

**Affiliations:** 1 St Vincent’s University Hospital, Elm Park, Dublin, Ireland; 2 UCD Centre for Interdisciplinary Research Education and Innovation in Health Systems (UCD IRIS), University College Dublin, Dublin, Ireland; 3 UCD School of Nursing, Midwifery and Health Systems, University College Dublin, Dublin, Ireland

**Keywords:** Aphasia, communication, decision-making, capacity, cognition, multidisciplinary team, functional capacity assessment

## Abstract

Formal assessments of cognition that rely on language may conceal the non-linguistic cognitive function of people with aphasia. This may have detrimental consequences for how people with aphasia are supported to reveal communicative and decision-making competence. This case report demonstrates a multidisciplinary team approach to supporting the health and social care decision-making of people with aphasia. The case is a 67-year-old woman with Wernicke’s type aphasia. As the issue of long-term care arose, the speech and language therapist used a supported communication approach with the patient who expressed her wish to go home. A multidisciplinary team functional assessment of capacity was undertaken which involved functional assessments and observations of everyday tasks by allied health, nursing, catering and medical staff. In this way, the patient’s decision-making capacity was revealed and she was discharged home. A collaborative multidisciplinary team approach using supported communication and functional capacity assessments may be essential for scaffolding the decision-making capacity of people with aphasia.

## Introduction

Following the United Nations Convention on the Rights of Persons with a Disability (UNCRPD), many states have introduced a legislative basis for the rights of all individuals, including those with a disability to be supported to participate as fully as possible in all decisions which affect their lives.^1^ In Ireland, the Assisted Decision-Making (ADM) (Capacity) Act was enacted in 2015. The Act introduced a statutory presumption that all individuals have decision-making capacity as well as a framework of tiered supports appropriate to different levels of functional decision-making capacity.^2^ The legislation provides a legal framework to support the decision-making of all patients, including those with a disability, ensuring their will and preferences are at the centre of all decisions about their health and social care.^3^

Across the literature, several different terms are used such as ‘supported decision making’ or ‘active decision making’. This article uses the term ADM as outlined in the Irish Act. In Ireland, as in other jurisdictions, legislative and policy innovations in response to the ratification of UNCRPD have proved challenging for implementation in health and social care settings.^4–7^ The ADM Act places an onus on Health and Social Care Professionals (HSCPs) to use supports that would scaffold the functional capacity of an individual to assist their healthcare decision-making. Overcoming communication barriers to accurately determine capacity and identify the appropriate supports for decision-making is a significant challenge for HSCPs. This is particularly relevant in cases of aphasia, an acquired neurological condition typically arising from a stroke. In Ireland, approximately 10,000 people per year have a stroke, and currently, 30,000 people are living in Ireland with stroke-related disability.^8^

Wernicke’s aphasia typically occurs post-stroke in approximately 16%–20% of patients in the acute phase, and in about 5% of cases it will persist at a chronic level.^9,10^ Alongside difficulties in understanding spoken language, Wernicke’s aphasia is often characterised by fluent speech with intact intonation, the presence of neologisms (nonsensical words) and a lack of content words. This makes it difficult for the listener to understand what the person with aphasia (PWA) is saying.^10^ Also affected is the person’s ability to recognise their speech errors and to self-correct when communication breaks down.^11^ This can result in frustration for the PWA if they are not understood or if the listener assumes the patient is ‘confused’ which occurs frequently.^4^ Formal cognitive assessments or screens such as the Mini-Mental State Examination (MMSE) and the Montreal Assessment of Cognition (MoCA) are typically language loaded which inhibits a complete assessment of cognition in PWA.^12,13^ They do not allow for the provision of appropriate support to facilitate communication to complete the test. This will result in misinterpretation of the decision-making abilities of PWA.^14^

The current case provides an example of a multidisciplinary approach to supporting the decision-making of a PWA. This patient-centred approach focuses on the scaffolding of decision-making capacity through the utilisation of relevant disciplinary expertise to support and reveal communication competence and a functional assessment of capacity. Functional assessment of language and communication allows the speech and language therapist (SLT) to observe how the PWA uses residual language skills. Assessment of functional capacity identifies the person’s ability to carry out essential tasks as a whole and evaluates their current level of functioning through observation of the strategies they use to complete tasks such as habits, routines, and contextual and environmental aids.^14^ This functional assessment was combined with a supported conversation approach (SCA) which revealed communication competence. SCA was developed by Aura Kagan in the Aphasia Institute to educate communication partners on how to communicate effectively with the PWA.^15^ This case report demonstrates that using the expert skills and resources available within the multidisciplinary team (MDT), including supportive conversation techniques, can not only reveal the communicative abilities of a PWA but can scaffold their capacity to make decisions regarding their care thereby supporting their autonomy.

## Case report

A 67-year-old, well-educated and previously independent woman was admitted to our institution after experiencing a perforated diverticular abscess and Hartmann’s procedure while on holiday. In the perioperative period, 2 weeks post abdominal surgery, while abroad, she experienced a left middle cerebral artery infarct. Seven days later she was repatriated, to our institution. On presentation, the patient was mobile with no apparent physical sequelae of stroke. The presenting complaint was that this previously independent woman now appeared to be confused, disorientated, and agitated, with Wernicke’s aphasia. She had a background history of asthma, rheumatoid arthritis, previous alcoholic liver disease, depression, and diverticular disease. Upon repatriation, neurological imaging was repeated, confirming the recent left middle cerebral artery infarct. Carotid arteries were bilaterally non-stenosed, an echocardiogram was non-contributory, and no cardiac arrhythmia was identified.

The patient was admitted under the stroke team and received regular input from members of the stroke MDT including social work, physiotherapy, dietician, occupational therapy, and speech and language therapy. During her admission, she also received consultations from other specialities including neurology, psychiatry, rehabilitation, rheumatology, hepatology, microbiology and colorectal teams. Initial occupational therapy assessment revealed the patient as agitated and requiring assistance of one person to complete all activities of daily living. The patient was documented to be confused, with poor attention, initiation, planning and sequencing of tasks. It was thought that she had a severe cognitive impairment; however, formal cognitive assessments could not be performed due to aphasia. On initial speech and language therapy assessment, the patient presented with fluent spoken language consisting of jargon and neologisms with some information words evident.

Two weeks into the admission, the therapists noted that the patient began to recognise multiple members of staff and show signs of orientation within the hospital. However, the patient did not appear to understand spoken language and was unaware that her spoken language did not make sense. She became frustrated when people did not understand what she was saying or misinterpreted what she said. This frustration was manifested through agitation. For example, the patient was brought to the hospital chapel as she produced the word church at some point in a conversation. She became agitated when brought to the church and was immediately brought back to the ward. She was unaware of the severity of her aphasia. She did not engage with formal assessment of language as she felt this was beneath her – ‘I’m not stupid, very intelligent’. It was difficult for staff and the patient’s family to interact with her as she became irritated and aggressive when people did not understand her. She was also sensitive to being asked to complete tasks she felt were too simple or easy, for example, spoken word to picture matching tasks, following basic commands such as open your mouth, making a cup of tea.

**Figure 1.**
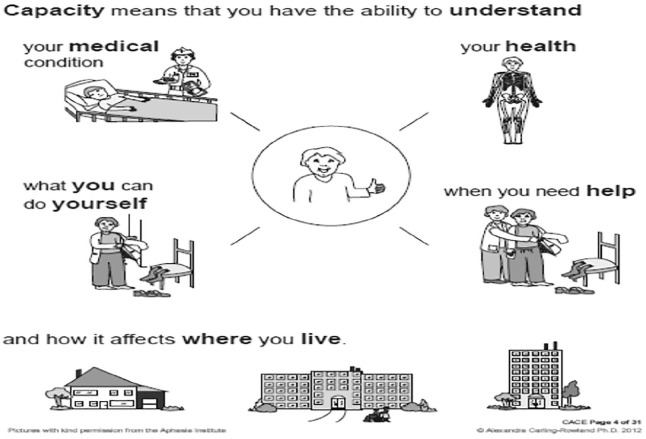
Sample A of communication tool used as part of the Communication Aid to Capacity Evaluation (CACE).

**Figure 2.**
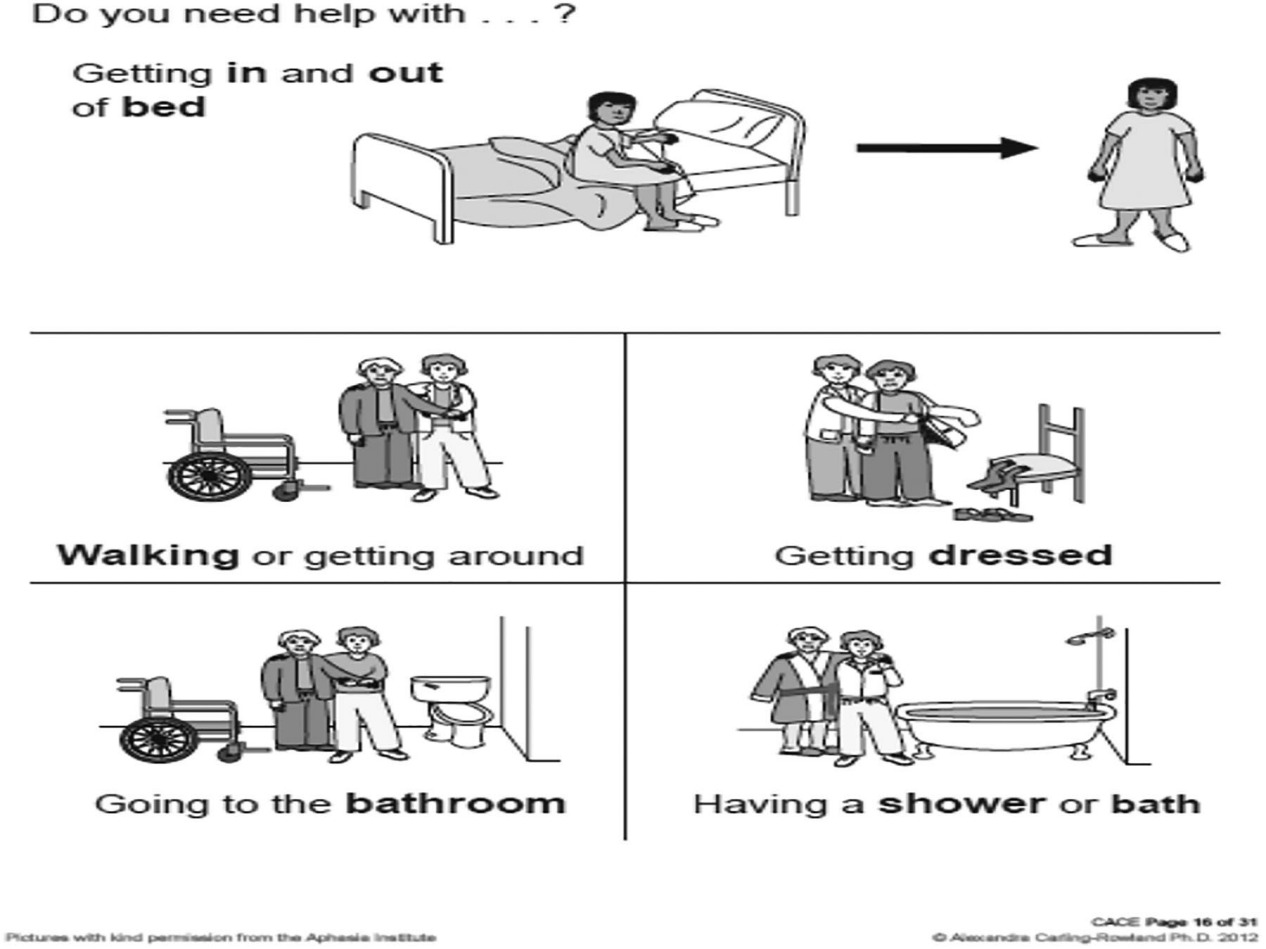
Sample B of communication tool used as part of the Communication Aid to Capacity Evaluation (CACE).

The issue of whether the patient had the capacity to make a decision regarding her discharge destination arose. Formal tests of cognition would not reveal true decision-making capacity due to the language required by the patient to complete them. It became clear that alternatives to these tests were required, particularly assessment approaches that focused on scaffolding functional capacity rather than cognition. Thus, a functional assessment of capacity was undertaken jointly by speech and language therapy and occupational therapy. Alternative assessment approaches included observation of functional tasks (e.g. making a meal), dynamic community tasks (e.g. trip to the local supermarket) and money management skills. Alongside these observations, The Communication Aid to Capacity Evaluation (CACE)^16^ was used to guide the SLT and occupational therapist (OT) on the patient’s understanding of her stroke and how it impacted her. The CACE was developed in 2012 purposively for adults who have a communication disability. It assesses orientation and ability to understand current care needs, proposed long-term care placement, present condition, the consequences of refusing long-term care placement and the consequences of accepting long-term care placement. This is all depicted in pictographic format for the PWA (see Figures 1 and 2). The CACE tool is appropriate for MDT collaboration as it is freely available, transferable across jurisdictions and is supported with free training for healthcare professionals. Using the CACE, it was revealed that she understood her present condition and how it impacted her and that her ultimate wish was to go home. This enabled the OT to discuss the need to assess her ability to complete activities of daily living which she agreed to.

Multidisciplinary (nurses, doctors) observations of the patient on the ward also offered insights into her cognition, communication and decision-making capacity. The team observed the patient in an unsupported communication environment where she was required to negotiate communication challenges arising from her not being understood. For example, she was noted as being competent to take on new learning when she worked with the clinical nurse specialist to learn how to change her colostomy bag independently.

**Figure 3.**
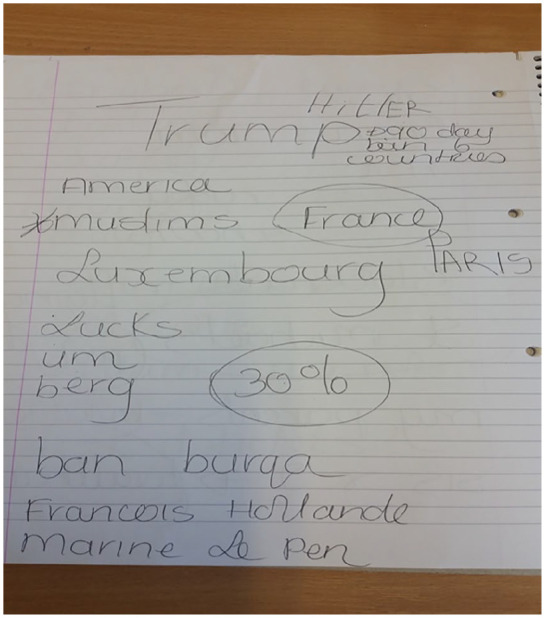
Illustration of complex conversation regarding politics using written choice communication.

Speech and language therapy focused on facilitating communication using an SCA.^15^ Visual aids, a personalised wordbook of key vocabulary (e.g. staff and family names, time, days of the week) to help the patient convey information, giving her time to respond, refocusing attention using tactile and visual cues, and written feedback on errors were employed to engage the patient in therapy and support functional communication. She responded well to this approach, creating her own personalised word book with the SLT by requesting her to write down staff and family names and relevant information about her care. It was possible to facilitate communication about novel information using her word book, maps and written multiple choices (see Figure 3). Over time it became apparent that, with support, the patient was a competent communicator and was gaining control over her care.

A functional approach to understanding and assessing capacity, SCA as well as MDT observations and discussions provided the scaffolding which supported the patient’s capacity to make her own decisions regarding her discharge. It was necessary to hold a family meeting with the patient and the MDT to facilitate discharge. The family were updated on progress to date and the patient’s ability to make decisions. The patient expressed her wish to go home. The family raised concerns about managing the patient’s behaviour when she was not being understood. Education sessions on supported communication were offered to MDT members as well as the family. Adjusting to new methods of communication was challenging for the patient’s family; however, they were keen and willing to learn how to facilitate conversation. A neuropsychological appointment was arranged with the patient and her husband, supported by the SLT, to help facilitate adjustment to the new way of communicating and the psychological changes experienced post-stroke.

The patient continues to live at home independently and was referred to community speech and language therapy for follow-up on her discharge. She continues to present with fluent aphasia but has adjusted well to the change in her communication. She continues to use verbal communication and a personalised communication book to communicate in all situations familiar and non-familiar.

## Discussion

Formal assessments of cognition that are language loaded, such as the MMSE, are inappropriate for assessing functional capacity and should never be used as part of an ADM approach to care planning with patients, particularly PWA.^12,17^ It is the role of the SLT to support PWA, to reveal not only their communicative abilities but also their decision-making capacity. Using supported conversation techniques such as visual aids/pictures, writing keywords and using simple straightforward language can not only reduce the linguistic load but also support cognitive processes required for decision-making by focusing the person’s attention and decreasing the memory load.^15^ Furthermore, multidisciplinary assessments of functional capacity evaluate the individual’s ability to perform activities of daily living which can provide HSCPs with information regarding metacognitive skills, executive function, motor skills and performance patterns.^18^ This information is important for a holistic assessment of function and decision-making capacity.

Strong interprofessional collaboration has been identified as a significant enabler of supported decision-making in health and social care settings for patients with complex needs.^5,19^ This requires a cultural change that will challenge hierarchical leadership within MDTs through the inclusion and empowerment of relevant specialist skills and disciplinary knowledge.^7^ This case report illustrates a collaborative multidisciplinary approach, with the utilisation of expert skills of relevant disciplines. This approach provided the necessary scaffolding to reveal the functional capacity of this patient. Meaningful MDT collaboration provided a holistic understanding of the patient’s capacity and ultimately supported her decision-making, thereby protecting her autonomy and dignity.

## Conclusion

In conclusion, a multidisciplinary approach that supports communication and focuses on a functional assessment of capacity and communication is an effective way to support decision-making for PWA and may be relevant to other populations too. The key learning points from this case are as follows:

- A multidisciplinary approach to assisted decision-making is key to revealing a person’s functional capacity to make decisions. Concerning PWA, it is essential to have an experienced SLT and OT on the team to lead the functional assessment of communication and capacity.
- It is important to explore variable avenues for communication, including communications aids. Scaffolding communication abilities through an SCA with different communication aids may be necessary to ascertain PWA’s will and preferences and to maximise their involvement in decisions that affect their lives.
- The patient who was previously irritated and refusing to engage with formal language assessment was enthusiastic about the supported communication approach. It became clear that with this support she was a competent communicator.
- Formal cognitive assessments are unsuitable for assessing decision-making capacity in PWA. A functional assessment of capacity using tools like the CACE, functional everyday tasks (e.g. making breakfast, shopping) behavioural observations and MDT discussions are essential for assessing and supporting the decision-making capacity of PWA.
- Every healthcare professional is responsible for supporting communication with their patient but particularly those presenting with aphasia.
